# Supplementary material for: Sequence imputation from low density single nucleotide polymorphism panel in a black poplar breeding population
Source: BMC Genomics. 2019 Apr 18;20:302. doi: 10.1186/s12864-019-5660-y (PMC6471894; doi:10.1186/s12864-019-5660-y)
Supplement: Supplementary file 2 — Relationship between the proportion of alleles correctly imputed by each leave-one-out individual (Propi) and the lower bound individual proportion of SNP correctly imputed lbPropi).The different colors correspond to the different individual classes in the mating regimes, and each point represents the values for one chromosome and one individual. (PDF 11.1 kb) [file 12864_2019_5660_MOESM2_ESM.pdf]

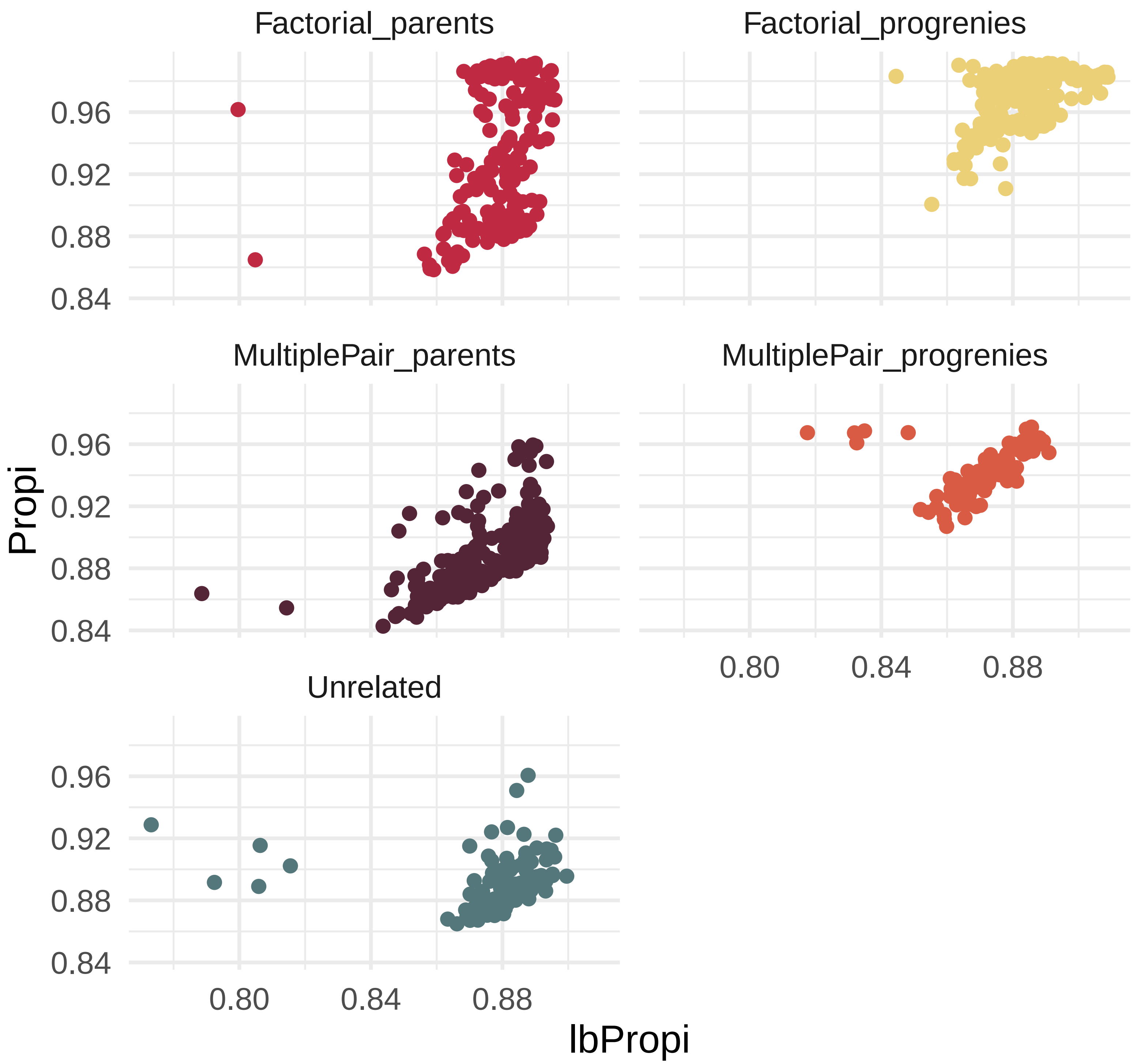

Factorial_parents
Factorial_progrenies
MultiplePair_parents
MultiplePair_progrenies
Unrelated
Propi
lbPropi
0.96
0.92
0.88
0.84
0.80
0.84
0.88
