# Supplementary figures and images for: Sequence imputation from low density single nucleotide polymorphism panel in a black poplar breeding population
Source: BMC Genomics. 2019 Apr 18;20:302. doi: 10.1186/s12864-019-5660-y (PMC6471894; doi:10.1186/s12864-019-5660-y)

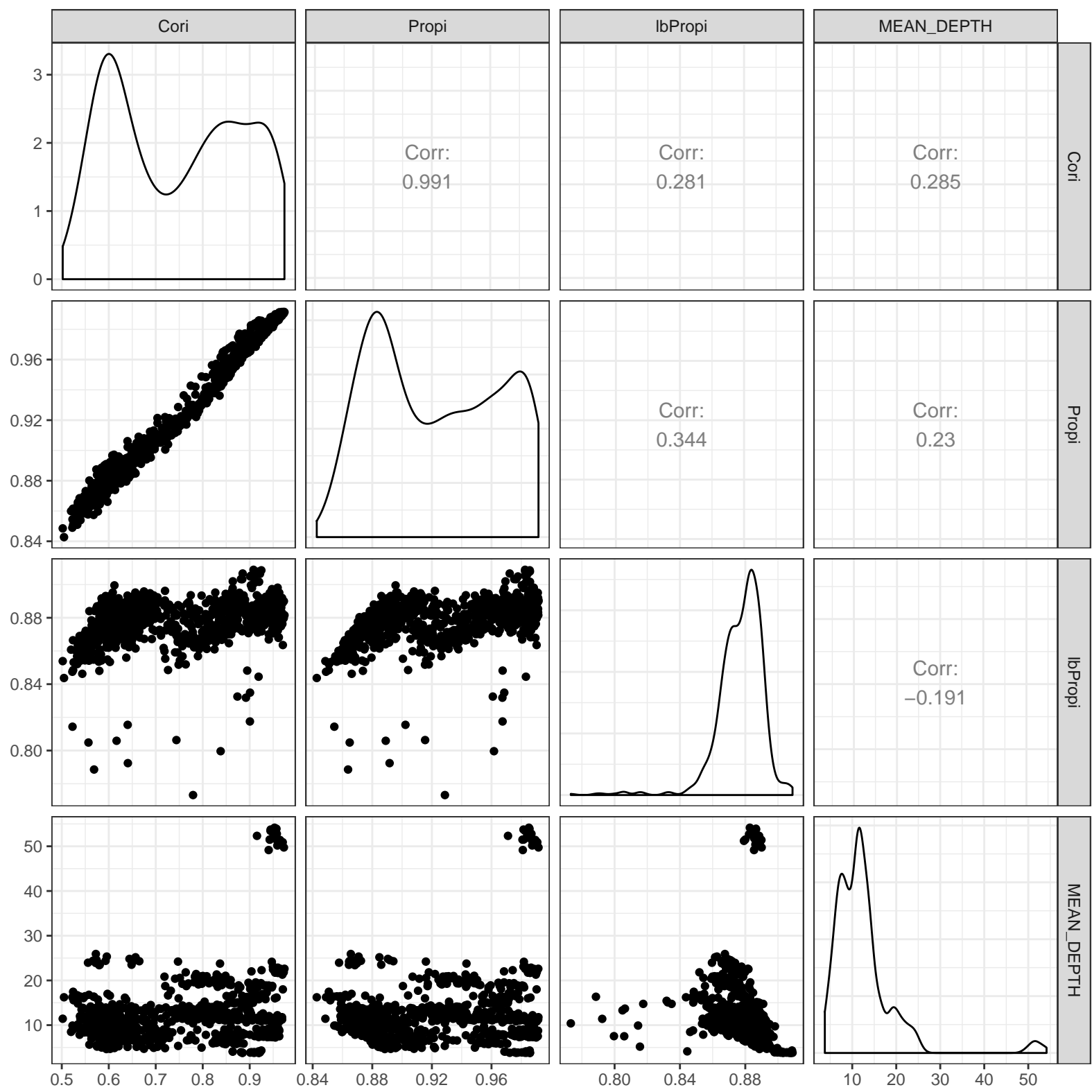

Supplement: Supplementary file 3 — Relationship between the sequencing depth and imputation quality variables at individual level. On the top of the diagonal: Pearson’s correlations. The distribution of each variable is shown on the diagonal. On the bottom of the diagonal: the bivariate scatter plots. (PDF 58.3 kb) [file 12864_2019_5660_MOESM3_ESM.pdf]

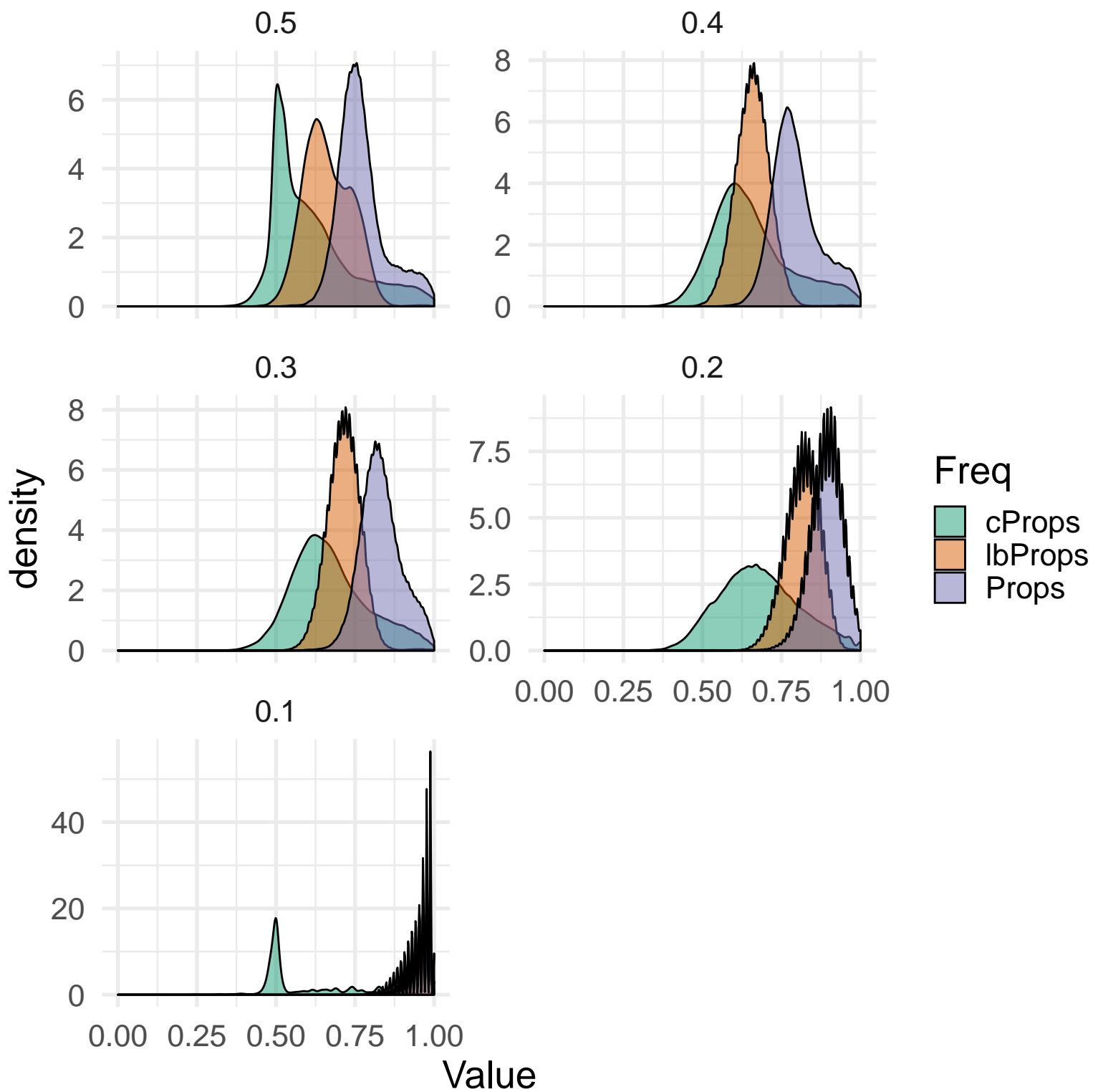

Supplement: Supplementary file 4 — Variation of the three different estimates of imputation quality at the SNP level (Props (Green), lbProps (Purple), cProps (Orange)), as a function of different classes of minor allele frequency (FreqOri). (PDF 42.3 kb) [file 12864_2019_5660_MOESM4_ESM.pdf]
